# Mechanistic Study of TTF-1 Modulation of Cellular Sensitivity to Cisplatin

**DOI:** 10.1038/s41598-019-44549-w

**Published:** 2019-05-29

**Authors:** Cody A. Phelps, Laura Lindsey-Boltz, Aziz Sancar, David Mu

**Affiliations:** 1 Leroy T. Canoles Jr. Cancer Research Center, Eastern Virginia Medical School, Norfolk, VA 23501 USA; 2 Department of Microbiology and Molecular Cell Biology, Eastern Virginia Medical School, Norfolk, VA 23501 USA; 3 Department of Biochemistry and Biophysics, University of North Carolina School of Medicine, Chapel Hill, NC 27599 USA; 4 Present Address: Carter Immunology Center, University of Virginia, Charlottesville, VA 22903 USA

**Keywords:** Cancer therapeutic resistance, Non-small-cell lung cancer

## Abstract

The lung lineage master regulator gene, Thyroid Transcription Factor-1 (TTF-1, also known as NKX2-1), is used as a marker by pathologists to identify lung adenocarcinomas since TTF-1 is expressed in 60 ~ 70% of lung ADs. Much research has been conducted to investigate roles of TTF-1 in lung cancer biology. But, how it modulates cellular chemosensitivity remains poorly characterized. Our study shows that TTF-1 sensitizes the *KRAS-*mutated A549 and NCI-H460 lung cancer cells to cisplatin, a common chemotherapy used to treat lung cancer. This chemosensitization activity does not appear to be mediated by a TTF-1-imposed alteration on nucleotide excision repair. Mechanistically, TTF-1 induced a reduction in p-AKT (S473), which in turn activated glycogen synthase kinase 3 (GSK3) and reduced β-catenin. Intriguingly, in the *EGFR*-mutated NCI-H1975 and HCC827 cells, *TTF-1* desensitized these cells to cisplatin; concomitantly, TTF-1 conferred an increase in p-AKT. Finally, the conditioned media of *TTF-1*-transefected cells sensitized *TTF-1*^−^ cells to cisplatin, implicating that the *TTF-1*-driven chemosensitization activity may be dually pronged in both intracellular and extracellular compartments. In short, this study highlights the enigmatic activities of TTF-1 in lung cancer, and calls for future research to optimally manage chemotherapy of patients with TTF-1^+^ lung ADs.

## Introduction

TTF-1 is a standard clinical marker of lung adenocarcinomas (ADs). The discoveries of *TTF-1* gene amplification in lung ADs provided the impetus to establish the functional roles of TTF-1 in lung tumorigenesis. Indeed, a plethora of functional consequences and downstream mediators of TTF-1 have been uncovered^1–13^. A consensus is that *TTF-1* exhibits either an oncogenic or an anti-tumorigenic role depending on the genetic context^14–16^. Intriguingly, prior to the documentation of *TTF-1* DNA copy number increases in lung ADs, multiple studies had established TTF-1 immunopositivity as an independent predictor of better survival in lung AD patients^17,18^. The anti-tumorigenic function of TTF- 1 seemingly aligns well with the trait of TTF-1 positivity being a better patient survival predictor. Yet, how TTF-1 may influence chemosensitivity of lung cancer cells and thus contribute to its prognostic power was not fully understood. Consequently, we initiated this study to glean a deeper mechanistic understanding of the connection between TTF- 1 and cellular chemosensitivity. There is evidence implicating chemosensitivity modulation by TTF-1 using *in vitro* cell systems. We reported an impact of the TTF-1 pathway activation on cisplatin sensitivity using a genomic approach^19^. Later, Liu *et al*. documented that TTF-1 ablation conferred carboplatin resistance in the *NRAS*-mutated NCI-H2087 lung cancer cells^5^, whereas Maeda *et al*. observed that Ttf-1 sensitized the A549 lung cancer cells to cisplatin^6^. Considering the fact the platinum compounds remain a widely used chemotherapy for lung cancer in the era of targeted and immunotherapies, we were particularly interested in understanding the mechanism of TTF-1-induced sensitization to cisplatin. In this study, we first reproduced the observation of TTF-1-dependent enhancement of cellular sensitivity to cisplatin. Since DNA damages induced by cisplatin are the major source of cytotoxicity^20^ and nucleotide excision repair (NER) is responsible for repairing such DNA lesions^21^, we investigated whether augmentation of NER contributed to the chemosensitivity reprogramming by TTF-1 and found no evidence. Motivated by the literature on the relationship between β-catenin and cisplatin sensitivity^22,23^, we determined that TTF-1 suppresses nuclear β-catenin likely via the canonical β-catenin destruction complex containing GSK3 because TTF-1 inhibits the kinase (AKT) that negatively regulates GSK3. These findings provide a deeper mechanistic understanding of the relationship between TTF-1 and cellular chemosensitivity. Although TTF-1 is a transcription factor that is not directly druggable by small molecules, studies such as the present one may shed new light on exploiting TTF-1 to improve lung cancer management. Case in point is our recent work which has identified TTF-1 as a putative indicator of lung cancer vulnerability to statins^24^.

## Results

### TTF-1 sensitizes A549 cells to cisplatin

**Figure 1:**
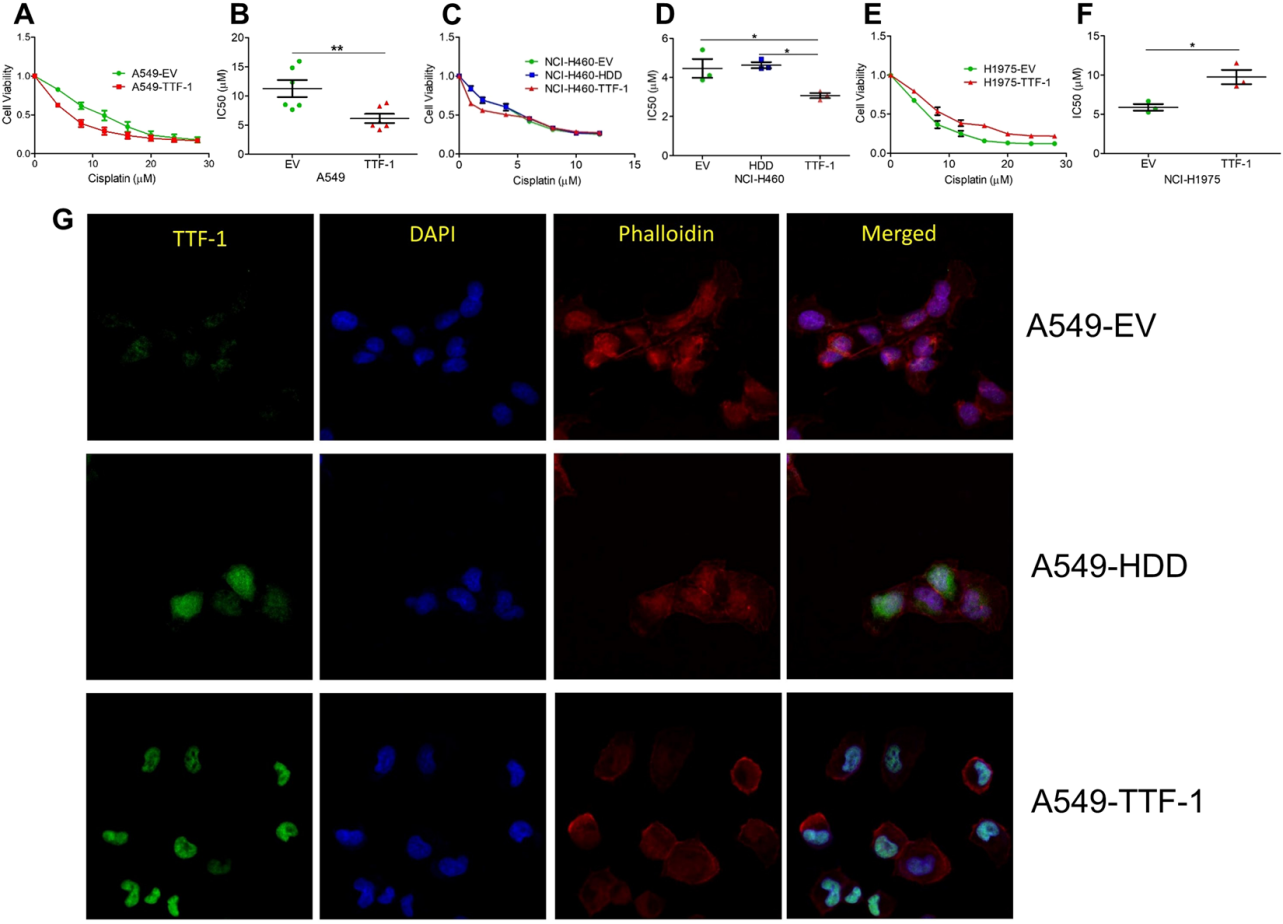
TTF-1 differentially sensitizes lung cancer cells to cisplatin. (**A**) Dose curves of A549-based transfectant cells (EV, or wt-TTF-1) treated with cisplatin. Data are the mean (±SEM) of 6 independent experiments. (**B**) IC50 values calculated from dose curves in A and two tailed t-tests were used to calculate statistical significance. (**C**) Dose curves of NCI-H460-based transfectant cells (EV, HDD, or wt-TTF-1) treated with cisplatin. Data are the mean (±SEM) of 3 independent experiments. (**D**) IC50 values calculated from dose curves in C and two tailed t-tests were used to calculate statistical significance. (**E**) Dose curves of NCI-H1975-based transfectant cells (EV or wt-TTF-1) treated with cisplatin. Data are the mean (±SEM) of 3 independent experiments. (**F**) IC50 values calculated from dose curves in E and two tailed t-tests were used to calculate statistical significance. Data shown in (**A–F**) were all derived from 48-hr cisplatin treatments. (**G**) A549 transfectant cells analyzed by confocal microscopy staining for TTF-1 expression (green), phalloidin (red), and DAPI (blue) at 63x magnification.

Intrigued by Maeda *et al*.^6^ reporting that the rat *Ttf-1* transgene sensitizes the *TTF-1*^−^ human lung AD cell line A549 to cisplatin, we investigated how the human *TTF-1* would impact cisplatin sensitivity of A549 cells. The expression of the *TTF-1* transgene in A549-TTF-1 cells was documented by immunoblotting and RT-QPCR in our published studies^24,25^. *TTF-1*-transfectant cells and a control - the empty vector (EV) transfectant cells – were treated with cisplatin for 48 hr (Fig. 1A). IC50 calculations implicated a significant increase (>80%) in cisplatin sensitivity of A549-TTF-1 cells (6.14 μM ± 0.78 μM SEM) in comparison with the EV control (11.28 μM ± 1.47 μM SEM, Fig. 1B). Next, we conducted dose-response analyses by subjecting cells to 72-hr cisplatin treatments. In these experiments, we included an additional control – cells transfected with the homeodomain deletion (HDD) mutant *TTF-1* which lacks DNA-binding activities^9^. Again, the *TTF-1* transfectant cells were more sensitive to cisplatin than the EV and HDD control cells (Fig. S1A,B). It is important to note that the *TTF-1* transgene did not significantly alter the proliferation of A549 cells (Fig. S1C). However, this TTF-1-dependent, heightened cisplatin sensitivity did not extend to another platinum chemotherapy – carboplatin (Fig. S2A,B); nor could we detect a TTF-1-dependent differential sensitivity toward gemcitabine (Fig. S2C,D), a chemotherapy sometimes used in combination with cisplatin for advanced nonsmall cell lung cancer (NSCLC)^26^. Therefore, we focused subsequent studies on cisplatin which is a standard chemotherapy for NSCLC^27^. It is interesting to note that the HDD mutant of TTF-1 did not appear to be localized in the nucleus as the wildtype protein was (Fig. 1G), based on the robust appearance of overlapping immunofluorescence of green (TTF-1) and blue (DAPI for DNA) only preferentially in the A549-TTF-1 cells (represented by the teal color). To substantiate this finding, we probed the expression of full-length TTF-1 and HDD mutant in the cytoplasmic and nuclear extracts of the A549 transfectant cells. The immunoblots demonstrated that while the full-length TTF-1 was exclusively associated with the nuclear extract, the HDD mutant was evidently present in cytoplasm (Fig. S3). These observations implicate the importance of the homeodomain for the nuclear localization of TTF-1.

### Diverse impacts of TTF-1 on cellular cisplatin vulnerability

Since A549 cells harbor a mutant *KRAS*^*G12S*^ allele, we next explored how a *TTF-1* transgene would impact the cisplatin sensitivity of another *KRAS* mutant-containing lung cancer cell line (NCI-H460, *KRAS*^*Q61H*^). Although NCI-H460 (referred to as H460) is more sensitive to cisplatin (IC50 ~ 4.5 μM) than A549, the *TTF-1* transgene decreased the cisplatin IC50 of H460 to 3.1 μM (a 31% decrease from 4.5 μM, Fig. 1C,D). In addition to *KRAS*, *EGFR* is another important lung oncogene whose mutation is mutually exclusive from the mutations of *KRAS* in lung adenocarcinomas^28^. To investigate how a *TTF-1* transgene would modulate cisplatin sensitivity under the *EGFR* mutant background, stable retrovirally transduced cells of NCI-H1975 (*EGFR*^*L858R*+^, referred to as H1975 hereafter) carrying either the wt-*TTF-1* transgene or the empty vector were analyzed for cisplatin sensitivity (Fig. 1E,F). Surprisingly, the *TTF-1* transgene desensitized H1975 cells to cisplatin, contrary to the observations based in A549 and H460 cells. We transfected *TTF-1* into another *EGFR* mutant lung cancer cell line (HCC827, *EGFR* exon 19 deletion) which is intrinsically more resistant to cisplatin than H1975 cells. Again, the *TTF-1* transgene desensitized the *EGFR*-mutated HCC827 cells to cisplatin (Fig. S4A,B). In a human lung cancer cell line that does not harbor either *KRAS* or *EGFR* mutations, i.e. NCI-H1437 (*TP53*^*R267P*^), intriguingly, *TTF-1* did not significantly alter its cisplatin sensitivity (Fig. S4C,D). An implication of these observations is that the cisplatin sensitivity modulation by TTF-1 may be genotype-dependent, warranting a larger study to examine this relationship. Nevertheless, the molecular mechanism of how TTF-1 confers cisplatin sensitivity in A549 cells remains to be determined and is the focal point of subsequent studies.

### TTF-1 does not influence cellular ultraviolet (UV) light sensitivity

**Figure 2:**
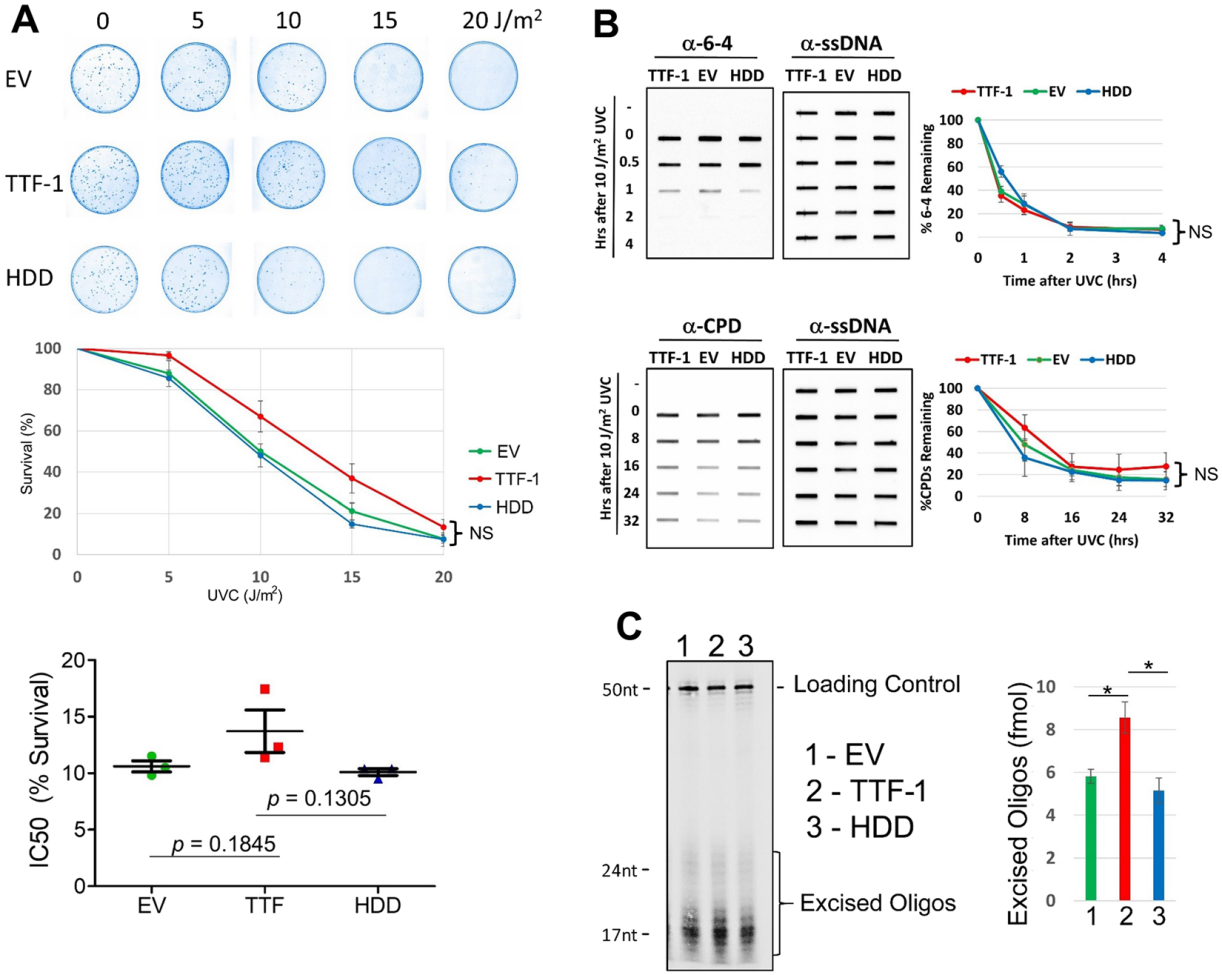
NER does not appear to mediate TTF-1’s modulation of cisplatin sensitivity. (**A**) Clonogenic survival assay (top), the quantified results (middle), and the IC50s of percent survival data (bottom) of the A549 transfectant cells. (**B**) Immunoslot blot repair assay to examine the repair kinetics of UV-induced DNA lesions in A549 transfectant cells. The graphs (to the right) contain the quantified data. (**C**) *In vivo* excision assay to compare the excised UV damage-containing oligonucleotides of the A549 transfectant cells. All experiments were conducted in triplicates. Graphed data are presented as (mean ± SEM). Two tailed t-tests were used to calculate statistical significance. NS, not significant.

DNA damages induced by cisplatin mediate cisplatin-induced cytotoxicity and are repaired by NER. A possible mechanism of TTF-1-dependent enhancement of cisplatin sensitivity is that TTF-1 reduces cellular NER activity. However, our observation of cellular carboplatin sensitivity not influenced by TTF-1 argues against this hypothesis. To substantiate this line of research, we assessed the UV light sensitivity of A549-based transfectant cells, in view of the fact that NER is the main mechanism to repair UV-induced DNA photoproducts in human cells^29^. The clonogenic survival assay results, as shown in Fig. 2A, demonstrated that there was no statistically significant difference in the UV light sensitivity between A549-TTF-1 cells and the two control cells. Using Immunoslot Blot assays^30^ to examine the repair kinetics of two major UV-induced DNA lesions (6–4 photoproducts and cyclopyrimidine dimers (CPDs)), we did not detect a significant difference in the repair of 6–4 photoproducts (Fig. 2B). However, the CPD repair appeared to trend slightly higher in the A549-TTF-1 cells. Finally, we quantified the damage-containing DNA oligonucleotides excised by NER *in vivo*^31^. The results again indicated the NER capacity was actually higher in A549-TTF-1 cells (Fig. 2C). These findings suggest that the TTF1-dependent enhancement of cisplatin sensitivity is unlikely mediated by a suppression of NER. Other TTF-1-dependent signaling events may be at play to heighten cellular vulnerability to cisplatin in the A549 cells.

### TTF-1 modulates β-catenin

**Figure 3:**
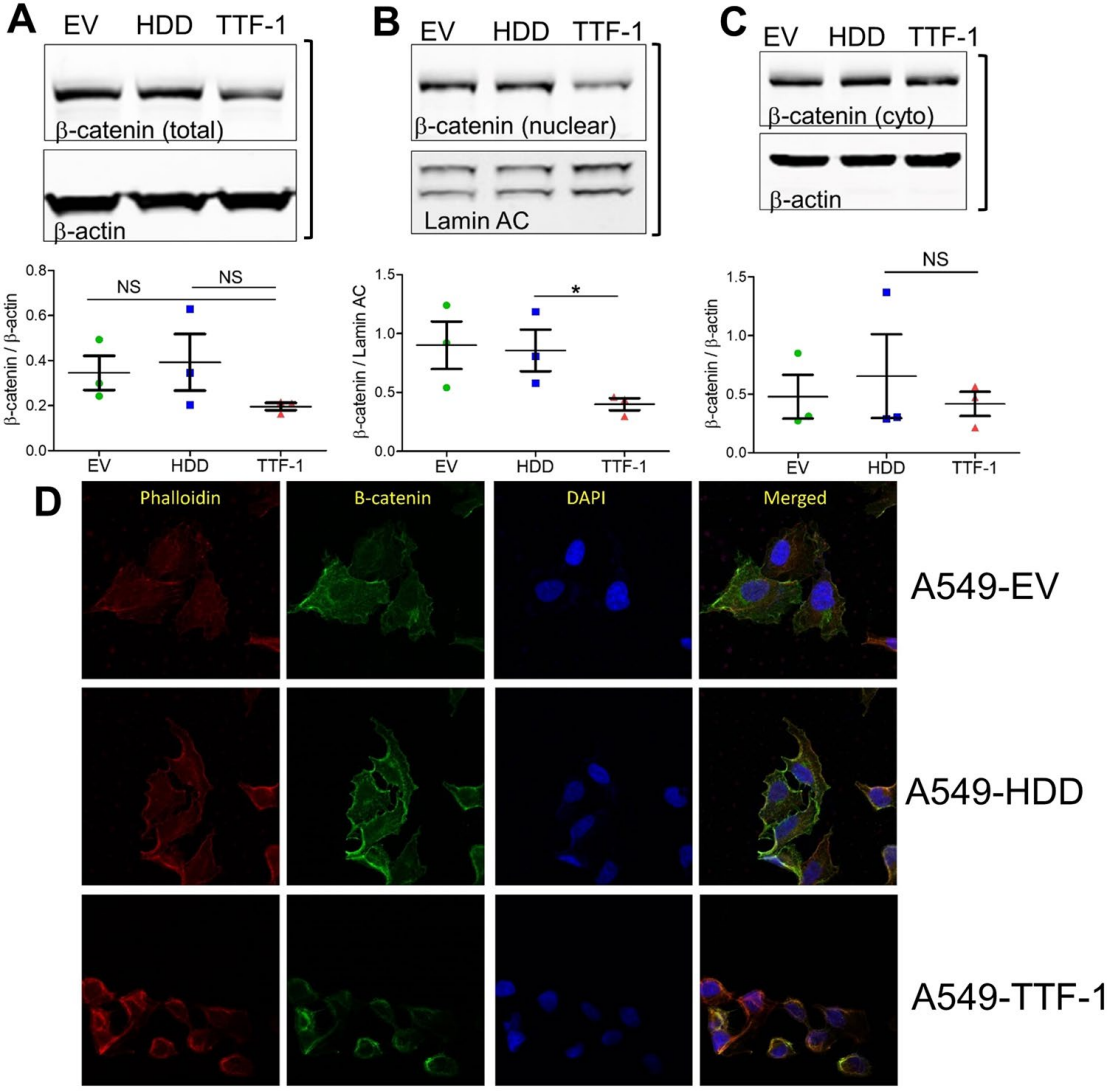
TTF-1 modulates β-catenin. Total (**A**), nuclear (**B**), and cytoplasmic (**C**) β-catenin was analyzed in the A549-derived cell lysates. Data are the mean (±SEM) of 3 independent experiments and one-way-ANOVAs with Tukey post hoc test were used to calculate statistical significance. (**D**) A549 cells analyzed by confocal microscopy staining for β-catenin expression (green), phalloidin (red), and DAPI (blue) at 63x magnification. The bracket next to the immunoblot images indicates the images were derived from the same gel.

Multiple considerations motivated us to examine β-catenin as a mechanistic lead mediating TTF-1-dependent enhancement of cisplatin sensitivity: (i) β-Catenin and TTF-1 functionally interact in the context of lung and thyroid development^32^, (ii) β-Catenin directly regulates *TTF-1* transcription^33^, and (iii) β-Catenin confers cellular cisplatin resistance^23,34^. We hypothesized that TTF-1 may feed negatively back to suppress β-catenin expression, which is expected to result in an enhancement of cisplatin sensitivity. To investigate how β-catenin expression may respond to a *TTF-1* transgene, we used immunoblotting to analyze three pools of β-catenin expression (total, nuclear, and cytoplasmic) in the A549-based transfectant cells. Interestingly, TTF-1 appeared to significantly reduce the nuclear presence of β-catenin (Figs 3A–C and S7), which was also supported by immunofluorescence analyzed by confocal microscopy, as β-catenin appeared to be more restricted to plasma membrane (Fig. 3D).

### The regulation of β-catenin by TTF-1 may involve GSK3

**Figure 4:**
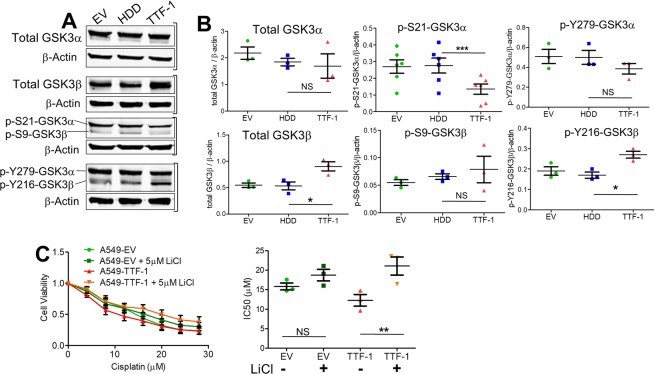
TTF-1 alters the phosphorylation of GSK3α/β. (**A**) Panels of immunoblot images examining total GSK3α/β and phosphorylation at specific Ser or Tyr residues in A549 transfectant cells. (**B**) Quantification of the immunoblots from independent experiments. Two tailed t-tests were used to calculate statistical significance. (**C**) *Left*: Dose curves of A549 cells transfected with TTF-1 or an empty vector (EV) control with or without LiCl (5 µM) to inhibit GSK3α/β. *Right*: IC50 calculations of dose curves. Two tailed t-tests were used to calculate statistical significance. The bracket next to the immunoblot images indicates the images were derived from the same gel.

Constitutively active GSK3 is a key component of the β-catenin destruction multiprotein complex, which targets Wnt effector β-catenin for phosphorylation, ubiquitination, and destruction^35,36^. Regulation of the two GSK3 isoforms (α and β) is mediated by inhibitory serine phosphorylation (S21-GSK3α and S9-GSK3β) and by activating tyrosine phosphorylation (Y279-GSK3α and Y216-GSK3β)^37^. By immunoblotting, a decrease in the inhibitory p-S21 of GSK3α was detected in the *TTF-1* transfectant cells (Figs 4A,B and S8), implicating a putative activation of GSK3α. Concurrently, the total GSK3β and the activating p-Y216 of GSK3β appeared to increase in the *TTF-1* transfectant cells (Fig. 4A,B), suggesting a simultaneous stimulation of both GSK3 isoforms by TTF-1. Presumably, GSK3 activation underlies the TTF-1-triggered loss of nuclear β-catenin through the β-catenin destruction complex. To substantiate this finding, we used a well-validated GSK3 inhibitor (LiCl)^38,39^ to probe the system. The rationale was that LiCl would dampen the TTF-1-dependent activation of GSK3 which in turn should reverse the enhanced cisplatin sensitivity conferred by TTF-1. As shown in Fig. 4C, LiCl desensitized A549-TTF-1 cells to cisplatin as predicted. On the other hand, LiCl appeared to exert a limited effect on the cisplatin sensitivity of the control A549-EV cells whose GSK3 activity is lower than that in A549-TTF-1 cells. These data demonstrate the mechanistic importance of the GSK3/β-catenin axis in TTF-1-driven cisplatin sensitization.

### TTF-1 inhibits AKT

**Figure 5:**
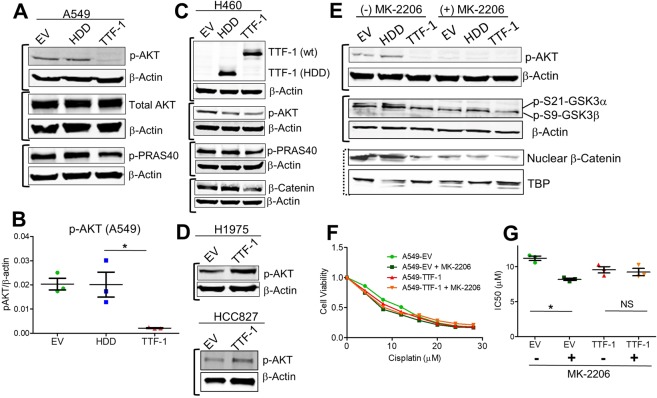
TTF-1 alters the phosphorylation and activation of AKT. (**A**) Panels of immunoblots examining total AKT, serine phosphorylation of AKT, phosphorylation of PRAS40, and β-actin in A549 transfectant cells. (**B**) Quantification of phosphorylation of AKT (Ser473) in A549 cells. One-way-ANOVAs with Tukey post hoc test were used to calculate statistical significance. (**C**) Panels of immunoblots examining TTF-1, total AKT, phosphorylation of AKT (Ser473), phosphorylation of PRAS40, and β-actin in NCI-H460 transfectant cells. (**D**) Immunoblots of NCI-H1975 and HCC827 transfectant cells for p-S473-AKT. (**E**) Immunoblots for p-S473-AKT, p-S21-GSK3α, p-S9-GSK3β, and nuclear β-catenin in the A549 transfectant cells with or without the AKT inhibitor MK-2206 (1 µM). (**F**) Cisplatin sensitivity assay of A549-EV and A549-TTF-1 cells with or without MK-2206 (1 µM). One-way-ANOVAs with Tukey post hoc test were used to calculate statistical significance. The solid bracket next to the immunoblot images indicates that the images were derived from the same gel. The dashed bracket next to the immunoblot images indicates that the images were derived from different gels of the same set of samples.

Multiple kinases, including AKT, can phosphorylate the inhibitory serines of GSK3^37^. We were particularly interested in AKT because a direct transcriptional target of TTF-1, i.e. receptor tyrosine kinase-like orphan receptor 1 (ROR1), acts functionally upstream of AKT^13^. Indeed, as reported by Yamaguchi *et al*.^13^, ROR1 was upregulated in A549-TTF-1 cells (Fig. S5). We therefore examined the AKT activation status, represented by p-S473, in the A549 based transfectant cells. Figure 5(A,B) shows that p-AKT was significantly depressed in A549-TTF-1 cells; concomitantly, a known downstream target of AKT (p-PRAS40, Proline-rich Akt substrate 40 kDa)^40^ was depressed as well in A549-TTF-1 (Figs 5A and S9). Importantly, in the H460 cell system, in which the *TTF-1* transgene also conferred cisplatin sensitization, the *TTF-1* transgene similarly inhibited p-AKT, p-PRAS40, and β-catenin (Figs 5C and S10). However, in the two *EGFR*-mutated cells (H1975 and HCC827) where the *TTF-1* transgene conferred cisplatin desensitization, the *TTF-1* transgene appeared to increase p-AKT (Figs 5D and S11). These observations are in line with the key finding presented in Fig. 4 that GSK3 activation (a known consequence of AKT deactivation) downregulates β-catenin and leads to cisplatin sensitization. To further corroborate these findings, we treated control and TTF-1 transfectant cells with a pan-AKT inhibitor (MK-2206)^41^. The anticipated outcome was that MK-2206 would phenocopy *TTF-1* in sensitizing A549-EV cells to cisplatin. As shown in Figs 5E and S12, MK-2206 obliterated p-AKT signals as expected. Moreover, MK-2206 further suppressed the inhibitory S9 phosphorylation of GSK3β, conferring a decline in the nuclear β-catenin levels of the two control A549 transfectant cells (EV and HDD, Fig. 5E). In terms of cisplatin sensitivity, it was as expected that MK-2206 treatments increased the cisplatin sensitivity of the A549-EV control cells (Fig. 5F,G). Overall, these data highlight the role of the AKT/GSK3/β-catenin in TTF-1-dependent cisplatin sensitization.

### The secretome of A549-TTF-1 cells also harbors the cisplatin sensitization activity

**Figure 6:**
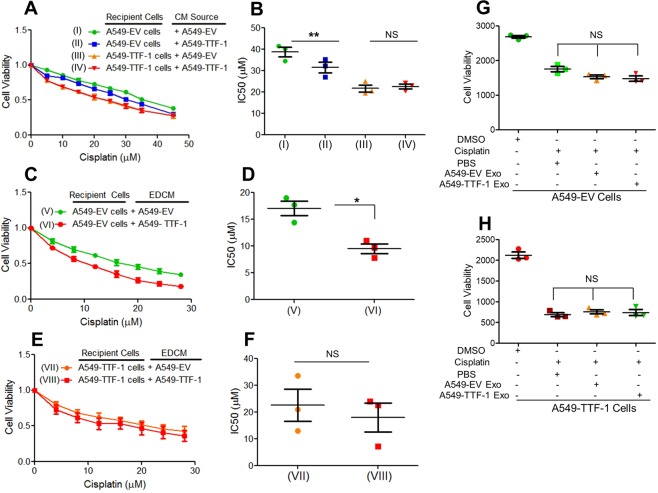
Conditioned media of A549-TTF-1 cells harbors cisplatin sensitizing effects. (**A**) Dose curves of cisplatin sensitivity assay. CM from 48-hr cultures of A549-EV or A549-TTF-1 cells was transplanted homotypically or heterotypically onto plated cells and incubated for 48 hrs. Data are the mean (±SEM) of 3 independent experiments (**B**) IC50 values calculated from dose curves in A and two tailed T-tests were used to calculate statistical significance. (**C**) Dose curves of cisplatin sensitivity of A549-EV cells receiving either A549-EV or A549-TTF-1 CM that has been exosome-depleted. (**D**) IC50 values calculated from dose curves in C and two tailed t-tests were used to calculate statistical significance. (**E**) Dose curves of cisplatin sensitivity of A549-TTF-1 cells receiving either A549-EV or A549-TTF-1 CM that has been exosome depleted. (**F**) IC50 values calculated from dose curves in E and two tailed t-tests were used to calculate statistical significance. (**G**) Cisplatin sensitivity assay of A549-EV cells in the presence of 10 µM cisplatin with or without exosomes harvested from A549-EV or A549-TTF-1 cells. (**H**) Cisplatin sensitivity assay of A549-TTF-1 cells in the presence of 10 µM cisplatin with or without exosomes harvested from A549-EV or A549-TTF-1 cells.

Our data so far establish mechanistically that the intracellular signaling axis (AKT/GKS3/β-catenin) mediates the cisplatin sensitization activity of TTF-1. In view of our published work that TTF-1 reprograms the secretome of lung cancer cells^25^, we hypothesize that the extracellular secretome, i.e. the conditioned media (CM), of A549-TTF-1 cells may also harbor the activity sensitizing naive A549 cells to cisplatin. To test this hypothesis, we used the CM harvested from A549-TTF-1 or the negative control cells (A549-EV) and transplanted them either homotypically or heterotypically onto recipient cells in the presence of increasing concentrations of cisplatin. Interestingly, we found that A549-EV cells incubated with the CM of A549-TTF-1 cells (denoted A549-TTF-1-CM) were more sensitive to cisplatin than the same cells incubated with A549-EV-CM (Fig. 6A,B). Conversely, similar media transplant experiments using the already sensitized A549-TTF-1 as the recipient cells didn’t reveal a further increase in cellular cisplatin sensitivity regardless of the type of CM imposed (Fig. 6A,B). To identify the CM compartment containing the cisplatin sensitization activity, we used differential ultracentrifugation to segregate CM into two compartments – (i) exosomes and (ii) exosome-depleted CM (EDCM). An exosomal marker (CD9)^42^ and nanoparticle tracking analysis were employed to characterize the exosome and EDCM fractions (Fig. S6). The cisplatin-sensitization activity appears to segregate with EDCM because A549-TTF-1-EDCM preferentially enhanced the cisplatin sensitivity of the control A549-EV cells but not that of A549-TTF-1 cells (Fig. 6C–F); whereas the exosomes, regardless of its source cells, did not alter the cisplatin sensitivity of either A549-EV or A549-TTF-1 cells (Fig. 6G,H).

## Discussion

**Figure 7:**
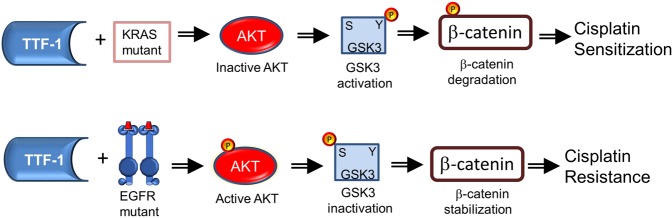
A schematic summarizing the key findings of this study. In the background of *KRAS* mutation, TTF-1 inactivates AKT, leading to GKS3 activation and β-catenin degradation. However, in the background of *EGFR* mutation, TTF-1 activates AKT, presumably leading to GKS3 inactivation and β-catenin stabilization.

How TTF-1 may impact cellular responses to cisplatin, a standard chemotherapy for lung cancer, was not well characterized. In the 2012 study by Maeda *et al*.^6^, it was shown that the rat *Ttf-1* transgene sensitized the human lung mucinous AD cell line (A549) to cisplatin. However, the molecular mechanism of this observation was not thoroughly investigated. In this study, we first reproduced the observation of Maeda *et al*. using the human *TTF-1* transgene. Next, we surmised that the impact of TTF-1 on cellular cisplatin sensitivity may be connected to NER in view of the report that TTF-1 interacts with Damaged DNA binding protein 1 (DDB1)^5^. DDB1 is part of a ubiquitin E3 ligase complex (DDB1-CUL4ADDB2) that initiates NER by recognizing damaged chromatin with concomitant ubiquitination of core histones at the lesion^43,44^. Thus, the known interaction between TTF-1 and DDB1 may compromise the NER-directed repair of cisplatin-induced DNA damages, which would presumably lead to a heightened sensitivity to cisplatin. However, by multiple DNA repair assays we did not obtain data supporting an involvement of NER in TTF-1-dependent regulation of cisplatin sensitivity. Therefore, we turned to β-catenin which is known to modulate cellular sensitivity to cisplatin^23,34^. The consideration that motivated us to investigate β-catenin was partially based on the documented positive regulation of E-cadherin by TTF-1 in A549 cells^45^. β-Catenin is involved in two apparently unrelated functions: (i) being part of a large protein complexes involved in E-cadherin-mediated cell adhesion, and (ii) providing the transcriptional activation domain within a nuclear complex with T cell factor family of transcription factors^46^. Thus, TTF-1-dependent regulation of E-cadherin would likely perturb β-catenin as well. Consistent with our hypothesis, TTF-1 reduced the levels of β-catenin in the nucleus. Since GSK3α/β are well-known regulators of β-catenin via the β-catenin destruction complex, we next analyzed various regulatory phosphorylations of GSK3α/β in response to TTF-1 (Fig. 4A,B). The specific alterations we detected included: (i) a reduction of inhibitory S21 phosphorylation of GSK3α, (ii) an increase of total GSK3β, and (iii) an increase of activating Y216 phosphorylation of GSK3β. Because only a small portion of β-catenin is associated with the GSK3-driven β-catenin destruction complex, it is known that the changes in nuclear β-catenin levels (rather than cytoplasmic or total nuclear β-catenin levels) are more reflective of β-catenin destruction complex activity^37^. Consequently, it is less surprising that only the nuclear β-catenin levels were significantly reduced by the TTF1-dependent upregulation of GSK3 activities in this study. Nevertheless, a corollary of our observations is that there appears to be a general activation of GSK3 by TTF-1. Importantly, the roles of GSK3 and β-catenin are further supported by the GSK3 inhibitor (LiCl) which dampened the cisplatin sensitization activity of TTF-1 (Fig. 4C). We then focused on AKT as it is one of the kinases that control GSK3 by phosphorylating GSK3 at the inhibitory serine residues, given the knowledge that ROR1, a downstream target of TTF-1, regulates AKT^13^. In both A549 and H460 cell lines harboring a mutant *KRAS* allele, *TTF-1* suppressed AKT activation which presumably unleashed GSK3 activation. Consistently, the AKT inhibitor MK-2206 sensitized the control A549-EV cells to cisplatin, mimicking TTF-1. In the two *KRAS* mutant backgrounds (A549 and H460), TTF-1 manifested the cisplatin sensitization phenotype. However, in H1975 and HCC827 cells which harbor *EGFR* mutations, TTF-1 elicited cisplatin desensitization. This is reminiscent of the known dimorphic phenotypes of Ttf-1 on tumorigenesis: being tumor-suppressive in the *Kras* mutant background^6,10,12^ but oncogenic in the *Egfr* mutant context^6^. Perhaps, how TTF-1 reprograms cellular chemosensitivity is also genotype dependent (Fig. 7). Future studies will be needed to solidify this concept.

In this study, we did not detect differential sensitivity at higher cisplatin concentrations for the NCI-H460 transfectant cells. This perplexing observation may be explained by the concept of different drug resistance mechanisms being deployed at high vs low concentrations of chemotherapies like cisplatin (active vs passive resistance per Stewart *et al*.^47^). The two control transfectant cells (EV and HDD) of NCI-H460 can be considered as having a shoulder on their dose‐response curve at low cisplatin concentrations (in comparison with the dose‐response curve of NCI-H460-TTF-1 cells). This shoulder may represent the “active resistance” caused likely by a saturable resistance mechanism that could be overloaded at higher concentrations of cisplatin. At higher cisplatin concentrations, this saturated resistance mechanism becomes inactive. Thus, the control cells are responding in the same way as the TTF‐1 transfectant cells.

Our observations of the intracellular signaling axis (AKT/GSK3/β-catenin) putatively mediating the cisplatin sensitization activity of TTF-1 does not preclude an involvement of TTF-1-directed extracellular events also contributing to the cisplatin sensitization phenotype. TTF-1 reprograms the secretome as evidenced by the work of Taguchi *et al*. who identified a protein signature of Ttf-1 in the plasma of mouse models of lung ADs^48^. Moreover, our recent study reported an alteration of secretome and exosomal cargos by TTF-1^25^. By using the CM of A549-TTF-1 cells to educate the control A549-EV cells, we showed that the CM of A549-TTF-1 sensitized the control A549-EV cells toward cisplatin. These observations implicate the presence of a TTF-1-driven extracellular component that contributes to cisplatin sensitization and raise intriguing mechanistic questions such as whether the CM of TTF-1^+^ cells decreases nuclear β-catenin levels in recipient cells and the potential roles of secreted Wnt inhibitory molecules (e.g., Dickkopf-related proteins) in the TTF-1-dependent phenotype of cisplatin sensitization. More research is needed to resolve these mechanistic questions and identify the culprit extracellular factors. In summary, this study provides the mechanistic insight on how TTF-1 increases cellular sensitivity to cisplatin, a chemotherapy commonly administered to lung AD patients. Given the fact that TTF-1 immunoreactivity is routinely analyzed for lung cancer patients, hopefully the results described herein inspire others to comprehensively investigate how TTF-1 modulates chemotherapeutic responses and translate the findings to improve lung cancer management.

## Methods

### Cell lines and reagents

A549, NCI-H460, NCI-H1437, NCI-H1975, and HCC827 cell lines were obtained from American Type Culture Collection. Cells were grown in RPMI-1640 (Corning Life Sciences 10-041-cv) in 10% FBS (VWR 89510-186) unless otherwise indicated. MK-2206 (Caymen Chemical, 11593), LiCl (VWR 97061-622). Cell lines were engineered to stably express TTF-1, TTF-1-HDD, or an Empty Vector (EV) control using retroviruses as previously described^7,49^.

### Chemosensitivity assays

Cells (3 × 10^3^) were plated in each well of a 96-well plate (Greiner bio-one 655090) with increasing concentrations of cisplatin (Cayman Chemical, 13119) dissolved in DMSO. DMSO concentrations were identical for all conditions. Each condition of cisplatin concentrations was performed in triplicate. After 48 or 72 hours, plates were read using a Cell Titer Blue reagent (Promega, G8081). IC50 calculations were obtained with Calcusyn software (Biosoft). Gemcitabine (Caymen Chemical, 11690) and carboplatin (TCI, C2043) experiments were done similarly. For experiments involving exosomes, 350 million exosomes were used per reaction. For assays with inhibitors, 1 µM of MK-2206 or 5 µM of LiCl was utilized based on Meng *et al*.^50^ and Coghlan *et al*.^51^, respectively.

### Immunofluorescence

Cells (1 × 10^4^) were plated into a well of an 8-well culture chamber slide (Falcon 354118) and incubated for 36 hours. Afterwards, cells were washed with PBS and blocked (0.3% Tritin X100, 1% BSA, 0.5% Normal Goat Serum in PBS). Primary antibodies were added for 75 minutes for β-catenin (Cell Signaling Technology, 8480S), TTF-1 (Santa Cruz Biotechnology, sc-13040), phalloidin-TRITC (Sigma Aldrich, P1951). Alexa-488 conjugated secondary goat-anti-rabbit antibody (Jackson ImmunoResearch, 111-545-045) was added after PBS wash for 60 min. After a final wash, mounting media with DAPI (Biotium, 23004) was added and a cover slip placed.

### Immunoblotting

Cell lysates were harvested with RIPA Buffer (150 mM NaCl, 5 mM EDTA, 50 mM Tris, 1% NP-40, 0.5% Sodium Deoxycholate, 0.1% SDS, 1X Halt Protease Inhibitor, 1 mM Na_3_VO_4_) and centrifuged for 30 min at 13,000 RCF and supernatant collected. The protein was separated using a 12% agarose gel and transferred to a nitrocellulose membrane. Antibodies for TTF-1 (Santa Cruz Biotechnology, sc-13040), β-catenin (Cell Signaling Technology, 8480S), total GSK-3α (Cell Signaling Technology, 4337), total GSK-3β (Cell Signaling Technology, 12456), Phospho-GSK-3α/β (Ser21/9) (Cell Signaling Technology, 8566), Anti-GSK3 (alpha + beta) (phospho Y216 + Y279) (abcam, 68476), pAkt (Cell Signaling Technology, 4060 S), Akt (Cell Signaling Technology, 4691 T) pPRAS40 (Cell Signaling Technology, 13175 T), PRAS40 (Cell Signaling Technology, 2691 T), β-actin (Novus, NBP1-47423), laminin (Cell Signaling Technology, 4777), and TBP (Biolegend, 668306) were used. Secondary antibodies (Licor, 926-32211, 926-68070) were used and read on a Licor Odyssey scanner. For nuclear and cytoplasmic separation, cells were rinsed with cold PBS and trypsinized, and centrifuged and washed with PBS three times. Cells were resuspended in cold Buffer 1 (10 mM HEPES, 10 mM KCl, 0.1 mM EDTA, 0.1 mM EGTA, 1 mM DTT, 1X Halt Protease inhibitor) for 15 minutes. Cells were lysed with NP-40 (final volume 0.6%) and inverted. Nuclei were pelleted at 10,000 g for 5 min. The supernatant represents the cytoplasmic fraction. The nuclei was resuspended in cold Buffer 2 (20 mM HEPES, 0.4 M NaCl, 1 mM EDTA, 1 mM EGTA, 10% glycerol, 1 mM DTT, 1X Halt Protease inhibitor) and lysed by shaking at 4 °C for 30 min. Lysates were centrifuged at 12,000 × g for 10 min at 4 °C.

### Conditioned Media and exosomes

Cells were plated and allowed to grow for 48 hours in RPMI-1640 with exosome depleted 1% FBS. The conditioned media was collected and centrifuged at 500 × g for 10 min to remove detached and dead cells. The supernatant was then collected and spun at 2,000 × g for 20 min to remove debris. The supernatant was again collected and spun at 20,000 × g for 30 min to remove larger microvesicles. The conditioned media was further centrifuged at 100,000 × g for 90 min. The supernatant represents the soluble fraction of the secretome (i.e., EDCM). The pellet was washed with an alkaline buffer (150 mM Na_2_CO_3_, pH 11) to break up protein aggregates and centrifuged again for 100,000 × g for 90 min. The exosome pellet was resuspended in PBS and kept at −80 °C. Quantification of the exosome concentration was performed using NTA (NanoSight NS300). For CM-based cisplatin sensitivity experiments, CM was collected as described above and was homotypically or heterotypically transplanted onto either A549-EV or A549-TTF-1 cells (replacing what would otherwise be fresh media) and allowed to incubate with cisplatin for 48 hrs. Cell viability was analyzed with cell titer blue as discussed above. The methods for conducting UV light sensitivity and NER assays are presented in the supplementary section.

### Statistical analysis

Quantitative experimental data were determined using biological replicates. Two-tailed t-tests and one-way ANOVAs were used to analyze data for statistical significance as described in the figure legends. *p ≤ 0.05; **p ≤ 0.01; ***p ≤ 0.001. P value > 0.05 is considered not significant.

## Supplementary information

Supplemental Methods and Figures

## Data Availability

All data generated or analyzed during this study are included in this published article (and its Supplementary Information files).
