# Supplementary material for: Mechanistic Study of TTF-1 Modulation of Cellular Sensitivity to Cisplatin
Source: Sci Rep. 2019 May 29;9:7990. doi: 10.1038/s41598-019-44549-w (PMC6541604; doi:10.1038/s41598-019-44549-w)
Supplement: Supplementary file 1 — Supplemental Methods and Figures [file 41598_2019_44549_MOESM1_ESM.pdf]

Supplementary Information

Mechanistic Study of TTF-1 Modulation of Cellular Sensitivity to Cisplatin

Cody A. Phelps[1,2] Laura Lindsey-Boltz[3], Aziz Sancar[3], and David Mu[1,2]

[1]Leroy T. Canoles Jr. Cancer Research Center, Eastern Virginia Medical School, Norfolk, VA 23501
[2]Department of Microbiology and Molecular Cell Biology, Eastern Virginia Medical School, Norfolk, VA 23501
[3]Department of Biochemistry and Biophysics, University of North Carolina School of Medicine, Chapel Hill, NC 27599

Supplementary Methods

Clonogenic Survival Assay (for UV sensitivity)

Cells were seeded in 10 cm plates at 300 cells/plate, grown for 16 hours, and then UV irradiated as follows. After removing and reserving culture medium, cells were placed under a GE germicidal lamp that emits primarily 254-nm UV light (UV-C) at 1 Joule m^{-2}/sec connected to a digital timer to give the indicated dose. Following irradiation, the culture medium was replaced, and the cells were incubated for 7 days. Plates were then washed with phosphate-buffered saline (PBS), the cells were fixed for 10 min in 75% methanol/25% acetic acid, and stained with 0.5% crystal violet stain (J.T. Baker) in 25% methanol for 30 min. The plates were then washed extensively in tap water to remove the excess stain. After drying, images of the stained cells were taken on a Molecular Imager Chemi-Doc XRS+ system (Bio-Rad) for presentation of representative, qualitative results. Cells were counted and the surviving fraction was calculated by dividing the number of colonies on treated plates by the number on the untreated plates. Each condition was performed in triplicate and the experiment was repeated three independent times.

Immunoslot Blot Analysis

Repair of UV photoproducts from genomic DNA was measured as described previously[1]. Cells were UV irradiated as described above for a dose of 10J/m^2. Following irradiation, the culture medium was replaced, and the cells were incubated for the indicated periods of time. The cells were washed with ice-cold PBS, harvested with a cell scraper, centrifuged at 1,600 × *g* in a microcentrifuge for 5 min at 4 °C, and then frozen on dry ice. Genomic DNA was isolated with a QIAamp DNA Mini kit (Qiagen). Genomic DNA (250 ng) was immobilized on a nitrocellulose membrane with a Bio-Dot SF Cell immunoslot blot apparatus (Bio-Rad) and heated for 90 min at 80 °C under vacuum. Blots were blocked in 5% milk in TBST (tris-buffered saline containing 0.1% Tween 20) and probed with an anti-(6-4)PP antibody (Cosmo Bio 64M-2 cat#NM-DND-001) or anti-CPD antibody (Cosmo Bio TDM-2 cat#NM-DND-002) as indicated. The secondary antibody was horseradish peroxidase-linked anti-mouse IgG from GE Healthcare (catalog no. NA931V). Chemiluminescent signals were visualized with Clarity Western ECL Substrate (Bio-Rad) and using a Molecular Imager Chemi-Doc XRS+ system (Bio-Rad). Following immunoblotting, the blots were re-blotted with anti-ssDNA antibody (Millipore MAB3034 clone 16-19) to ensure equal loading of DNA. The experiment was repeated three times, and representative results are presented.

Detection of Excised Oligonucleotide Products of Nucleotide Excision Repair

Nucleotide excision repair activity was visualized as previously described[2]. Cells grown to ~80% confluency in 15-cm plates were harvested 1 h after irradiation with 20 J/m^2 of UV. The cells were pelleted by centrifugation, subjected to a modified Hirt procedure where cell pellets were resuspended in a 10× packed cell volume of lysis buffer (50 mM Tris-Cl, pH 8.0, 10 mM EDTA, 1.2% SDS and 100 µg/ml RNase A) and incubated for 15 min at room temperature. Following addition of a one-fourth volume of 5 M NaCl, the mixtures were gently mixed and incubated on ice for 12–16 h. After centrifugation at maximum speed (20,000 × g) for 1 h, the supernatants were gently transferred to new tubes and treated with 20 µg of proteinase K for 15 min at 55 °C, extracted with phenol/chloroform, and then precipitated with ethanol. The pellet was washed with 500 µl of 70% ethanol and resuspended in 10 µl of buffer EB (10 mM Tris-Cl (pH8.5)). The

excised oligonucleotide products of nucleotide excision repair were purified from the remaining material with anti-(6-4)PP antibodies as follows: For each reaction, 5 µl of protein G Dynabeads (Invitrogen, catalog no. 10003D) slurry and 5 µl of anti-rabbit Dynabeads (Invitrogen, catalog no. 11203D) slurry were washed three times with 50 µl of wash buffer I (20 mM Tris-Cl (pH 8.0), 2 mM EDTA, 150 mM NaCl, 1% Triton X-100, and 0.1% SDS) and then incubated with 1 µl of rabbit anti-mouse IgG and 1 µl of anti-(6-4)PP antibody in 20 µl of IP buffer (20 mM Tris-Cl (pH 8.0), 2 mM EDTA, 150 mM NaCl, 1% Triton X-100, and 0.5% sodium deoxycholate) for 3 h at 4 °C. After incubation, beads were separated from the liquid with a magnet and then mixed with 100 µl of IP buffer and 10 µl of DNA. The mixtures were rotated at 4 °C overnight. The beads were then washed sequentially with 200 µl each of wash buffer I, wash buffer II (20 mM Tris-Cl (pH 8.0), 2 mM EDTA, 500 mM NaCl, 1% Triton X-100, and 0.1% SDS), wash buffer III (10 mM Tris-Cl (pH 8.0), 1 mM EDTA, 150 mM LiCl, 1% Nonidet P-40, and 1% sodium deoxycholate), wash buffer IV (100 mM Tris-Cl (pH 8.0), 1 mM EDTA, 500 mM LiCl, 1% Nonidet P-40, and 1% sodium deoxycholate) and finally twice with TE (10 mM Tris-Cl (pH 8.0) and 1 mM EDTA). The oligonucleotides containing UV photoproducts were eluted by incubation with 100 µl of elution buffer (50 mM NaHCO3, 1% SDS, and 20 µg/ml glycogen) at 65 °C for 15 min. The eluted DNA was then isolated by phenol/chloroform extraction and followed by ethanol precipitation. The excised oligonucleotides were resuspended in 10 µl of water, and half of the DNA was 3′-end labeled for 1 h at 37 °C in a 10-µl reaction containing 6 units of terminal deoxynucleotidyl transferase (New England Biolabs), 0.25 mM $CoCl_2$, and 1 µCi of [α-32P]-3′-deoxyadenosine 5′-triphosphate (cordycepin 5′-triphosphate, Perkin Elmer Life Sciences) in 1× terminal deoxynucleotidyl transferase buffer (New England Biolabs). 2.5 fmol of a 50-nucleotide oligomer was included in all reactions as an internal control, and oligonucleotides of known length were resolved on all gels as size markers. Following phenol-chloroform extraction and ethanol precipitation, the DNA was separated on urea-containing polyacrylamide gels, detected with a phosphorimager and was quantified using ImageQuant software (version 5.2, GE Healthcare). The experiment was repeated three times.

1 Gaddameedhi, S., Kemp, M. G., Reardon, J. T., Shields, J. M., Smith-Roe, S. L., Kaufmann, W. K. & Sancar, A. Similar nucleotide excision repair capacity in melanocytes and melanoma cells. *Cancer Res.* **70**, 4922-4930 (2010).

2 Hu, J., Choi, J. H., Gaddameedhi, S., Kemp, M. G., Reardon, J. T. & Sancar, A. Nucleotide excision repair in human cells: fate of the excised oligonucleotide carrying DNA damage in vivo. *J. Biol. Chem.* **288**, 20918-20926 (2013).

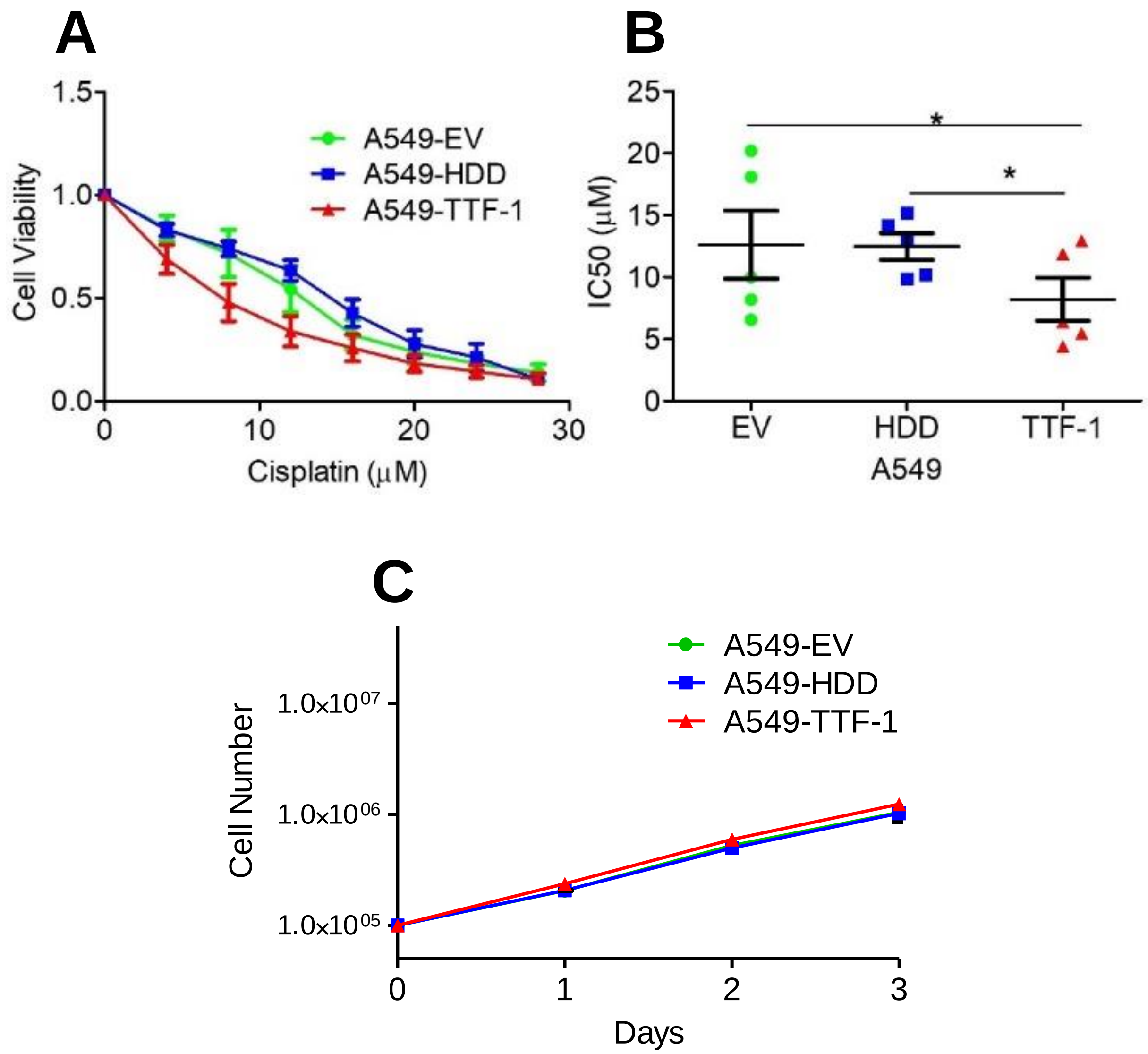

Figure S1. *TTF-1* sensitizes cells to cisplatin. (**A**) Dose curves of A549-based transfectant cells (EV, HDD, or wt-TTF-1) treated with cisplatin for 72 hr. Data are the mean (± SEM) of 5 independent experiments. (**B**) IC50 values calculated from dose curves in A and two tailed t-tests were used to calculate statistical significance. (**C**) Human *TTF-1* transgene does not significantly alter A549 cell proliferation. Cells were monitored for 3 days following plating in triplicates using a hemocytometer. No significant difference was detected by two-tailed t-test.

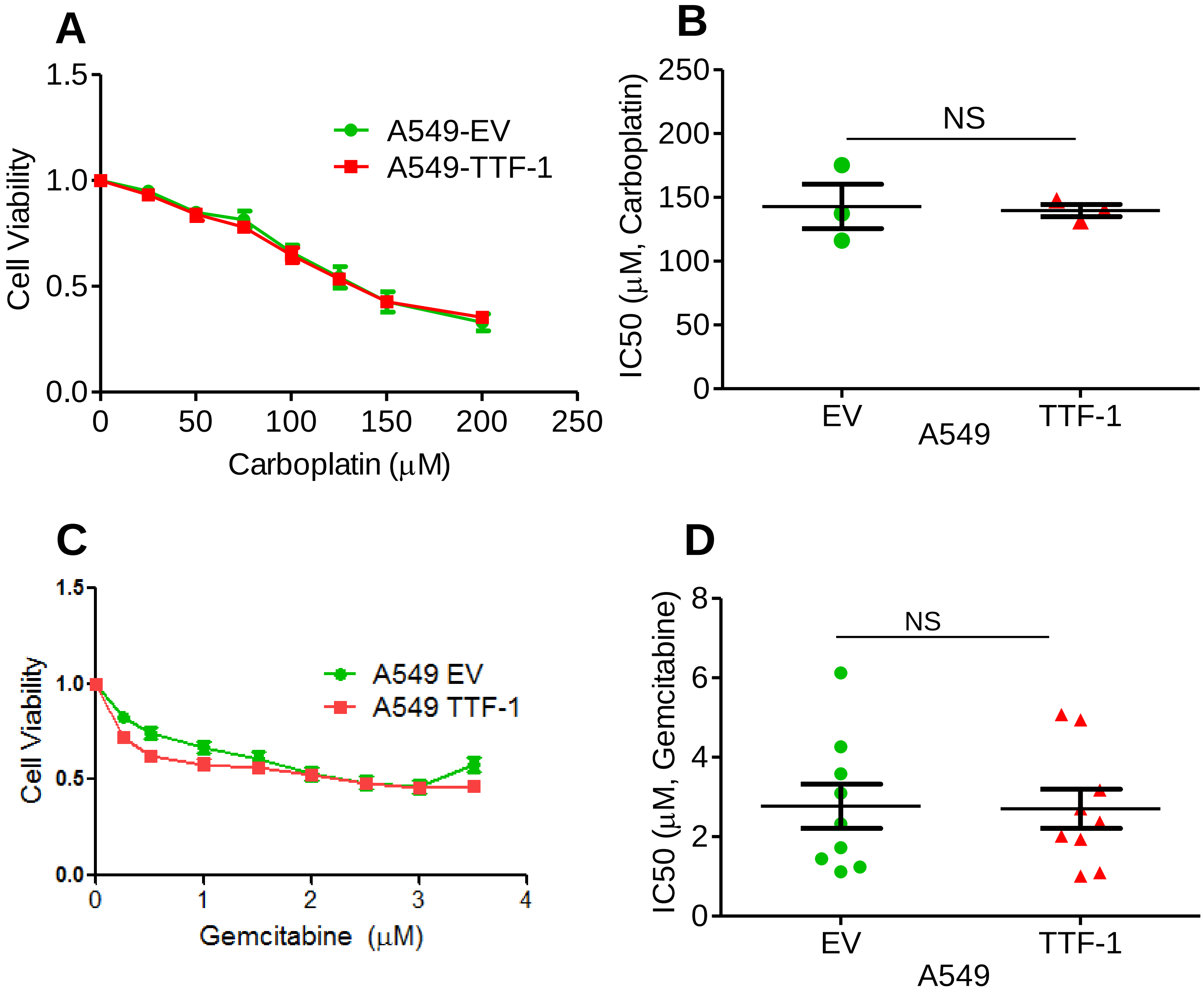

Figure S2. Human *TTF-1* transgene does not sensitize A549 cells to carboplatin or gemcitabine. (**A**) Dose-response data of A549 transfectant cells to carboplatin. (**B**) IC50s calculated from the dose-response data in A. (**C**) Dose-response data of A549 transfectant cells to gemcitabine. (**D**) IC50s calculated from the dose-response data in C. Cells were exposed to drug treatments for 48 hr. Data shown: mean ± SEM. NS, not significant. Two-tailed t-tests were used to calculate statistical significance.

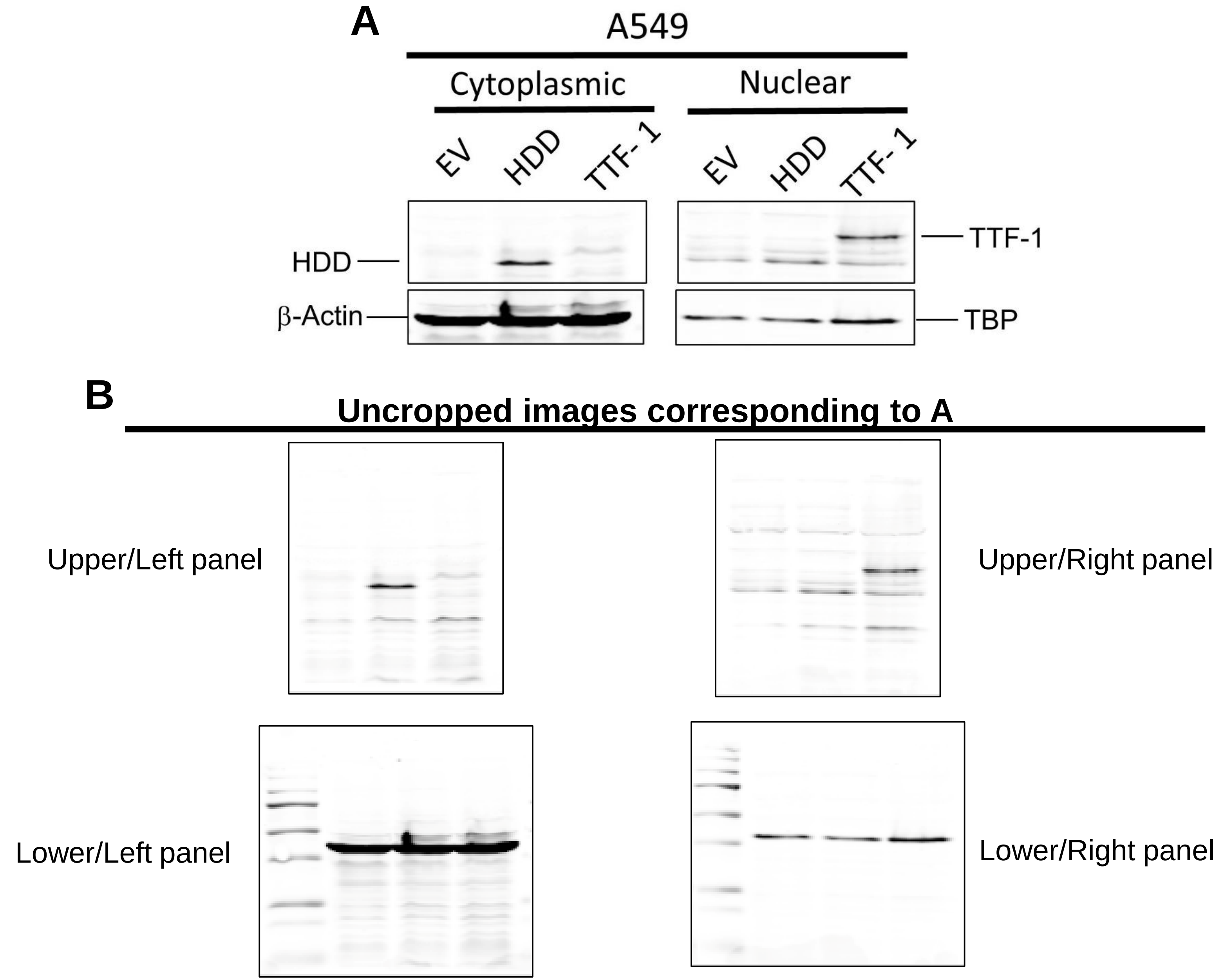

Figure S3. The HDD mutant of TTF-1 is present in the cytoplasm. (**A**) The cytoplasmic and nuclear extracts of the A549 transfectant cells were probed by the indicated antibodies. TBP, TATA-binding protein. (**B**) Original uncropped images of the four panels shown in A.

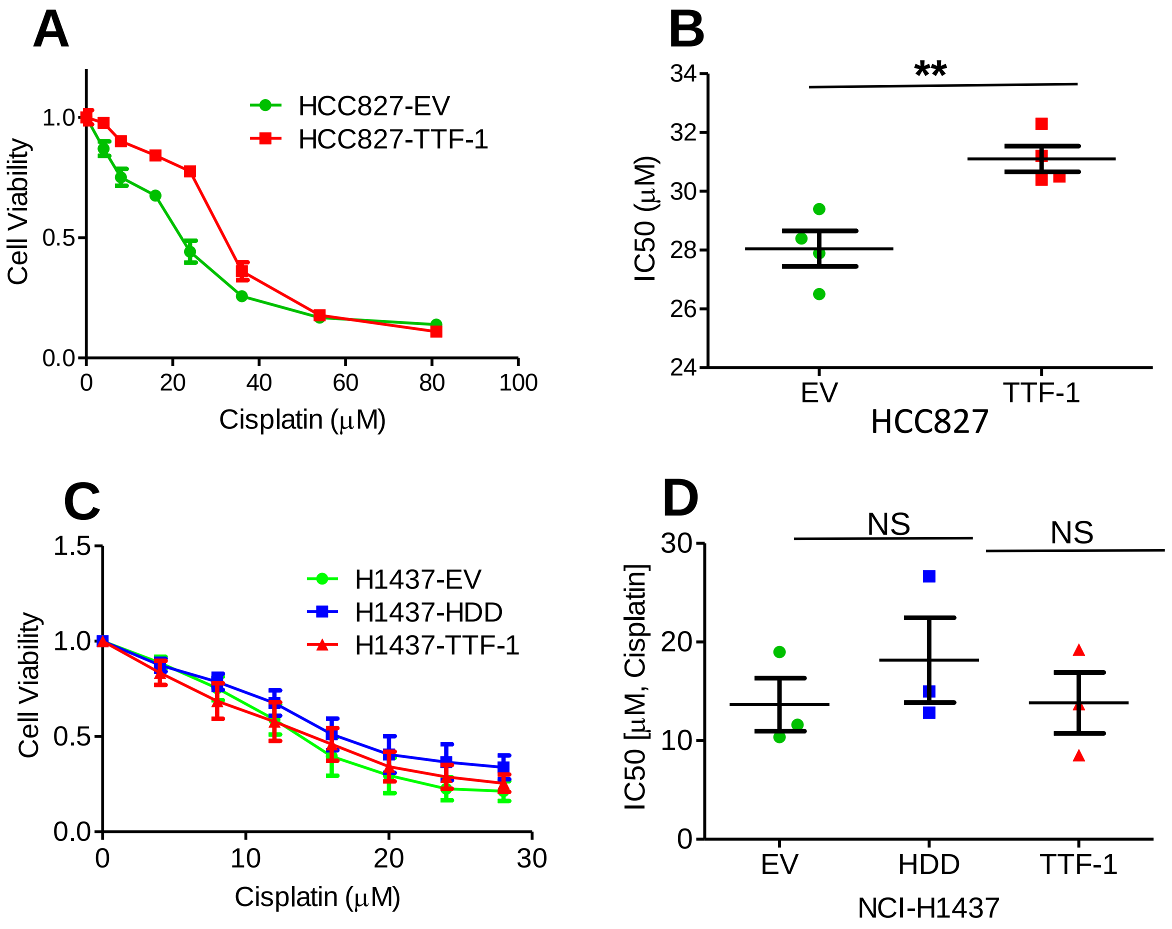

Figure S4. Human *TTF-1* transgene induces diverse cellular responses to cisplatin. (**A**) Dose-response data of HCC827 transfectant cells to cisplatin. (**B**) IC50s calculated from the dose-response data in A. (**C**) Dose-response data of NCI-H1437 transfectant cells to cisplatin. (**D**) IC50s calculated from the dose-response data in C. Cells were treated with cisplatin for 48 hr. Two-tailed t-tests were used to calculate statistical significance. Data shown: mean ± SEM. NS, not significant.

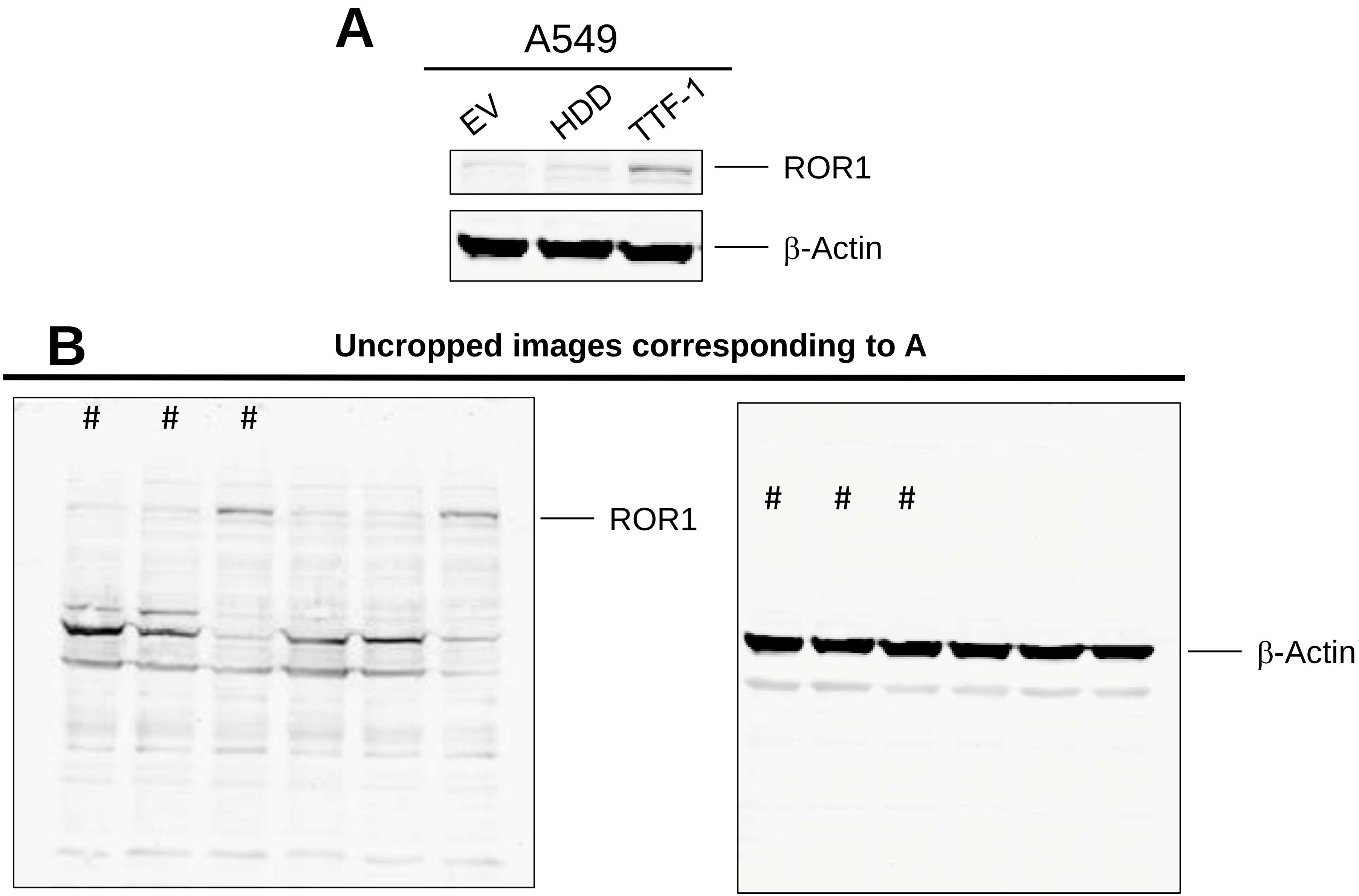

Figure S5. ROR1 is positively regulated by TTF-1. (**A**) Immunoblots show that ROR1 is upregulated in the A549-TTF-1 transfectant cells. (**B**) Uncropped images of immunoblots shown in A. # indicates the lanes containing the cropped bands shown in A.

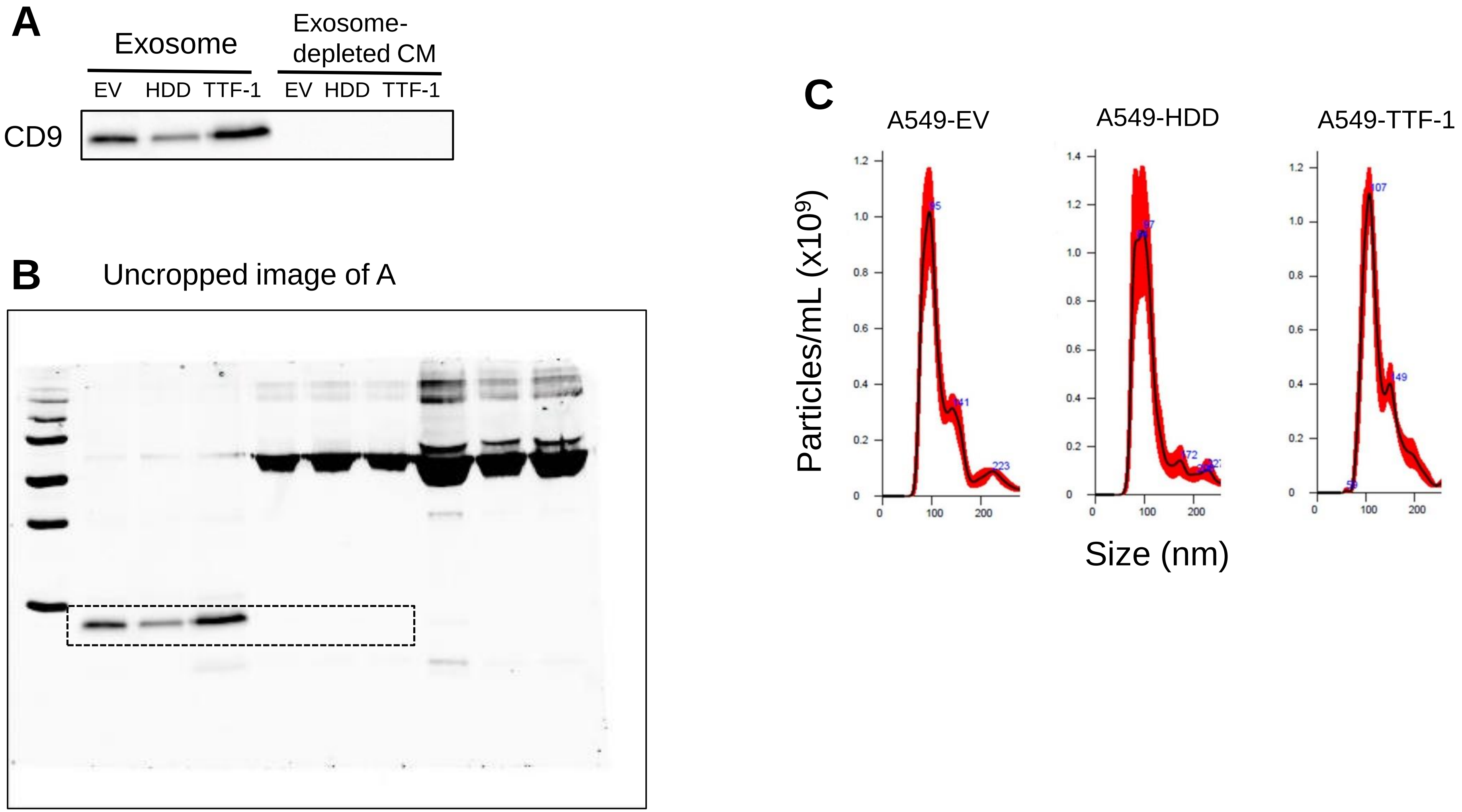

Figure S6. Exosome characterizations. (**A**) CD9 immunoblotting of exosomes and exosome-depleted CM of the A549 transfectant cells. (**B**) Original uncropped image of A. The frame indicates the cropped image shown in A. (**C**) Nanoparticle tracking analysis of the exosome size distributions.

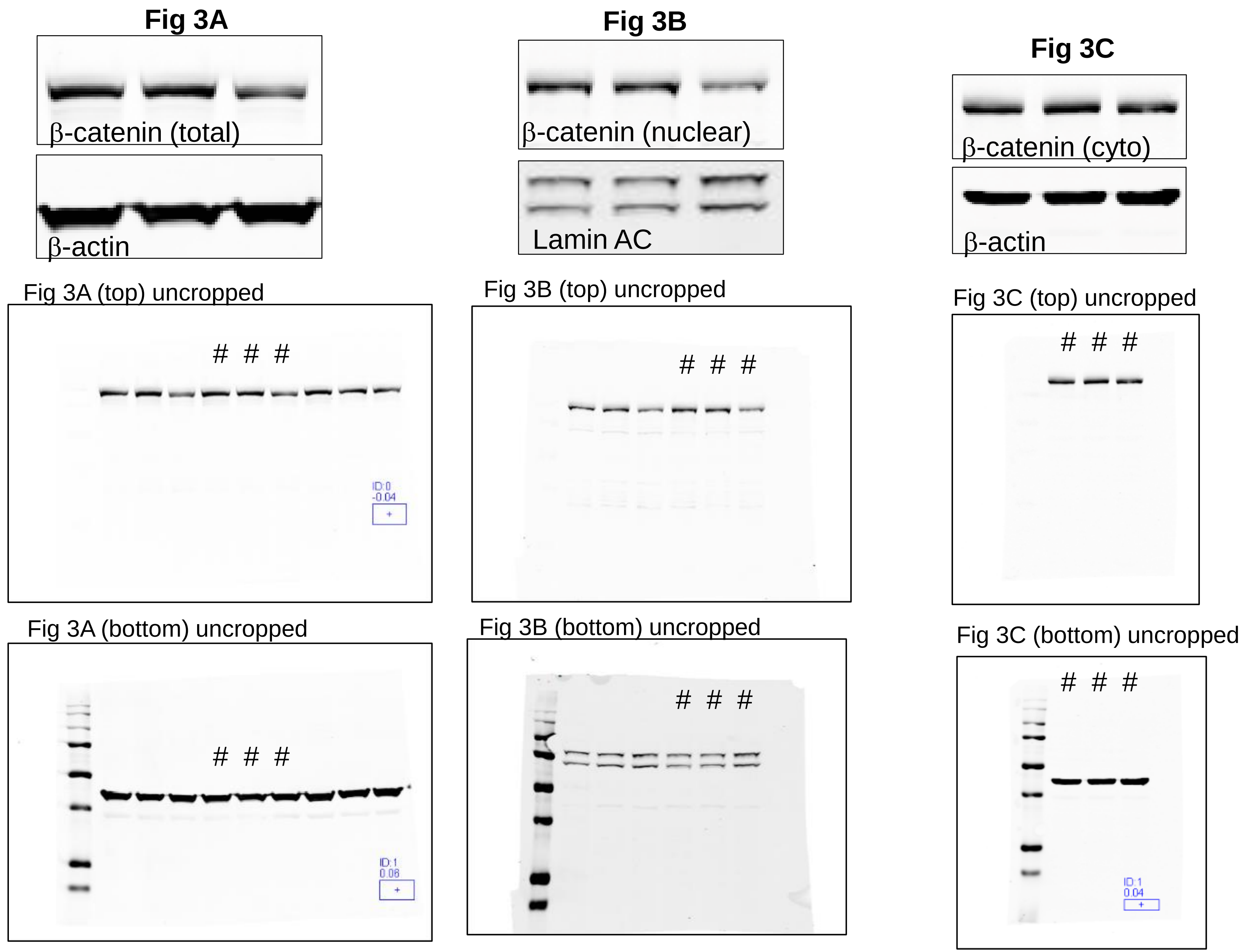

Figure S7. Fig 3 and the original uncropped images. # indicates the lanes containing the cropped bands shown in Fig 3.

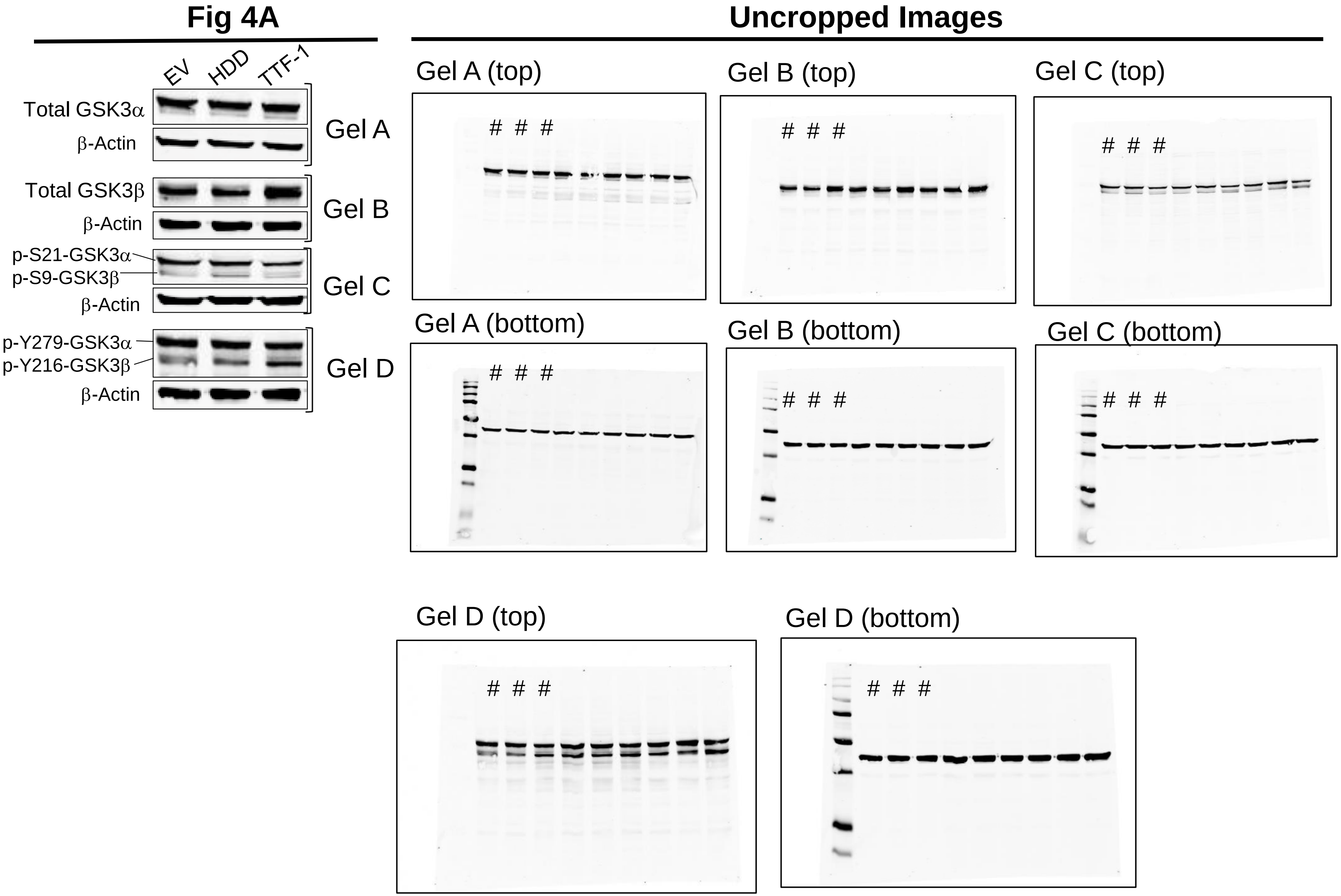

Figure S8. Fig 4A and the original uncropped images. # indicates the lanes containing the cropped bands shown in Fig 4A.

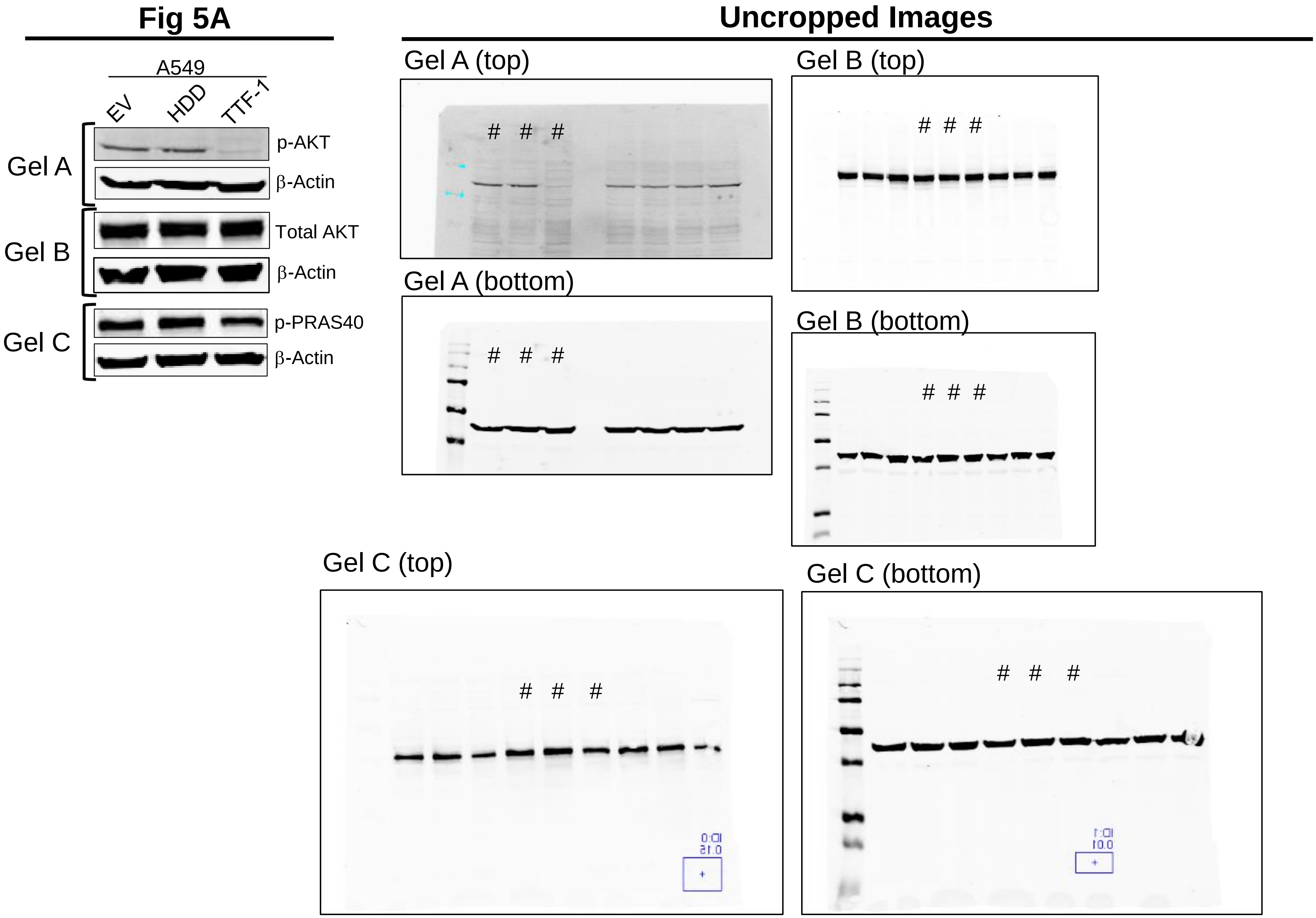

Figure S9. Fig 5A and the original uncropped images. # indicates the lanes containing the cropped bands shown in Fig 5A.

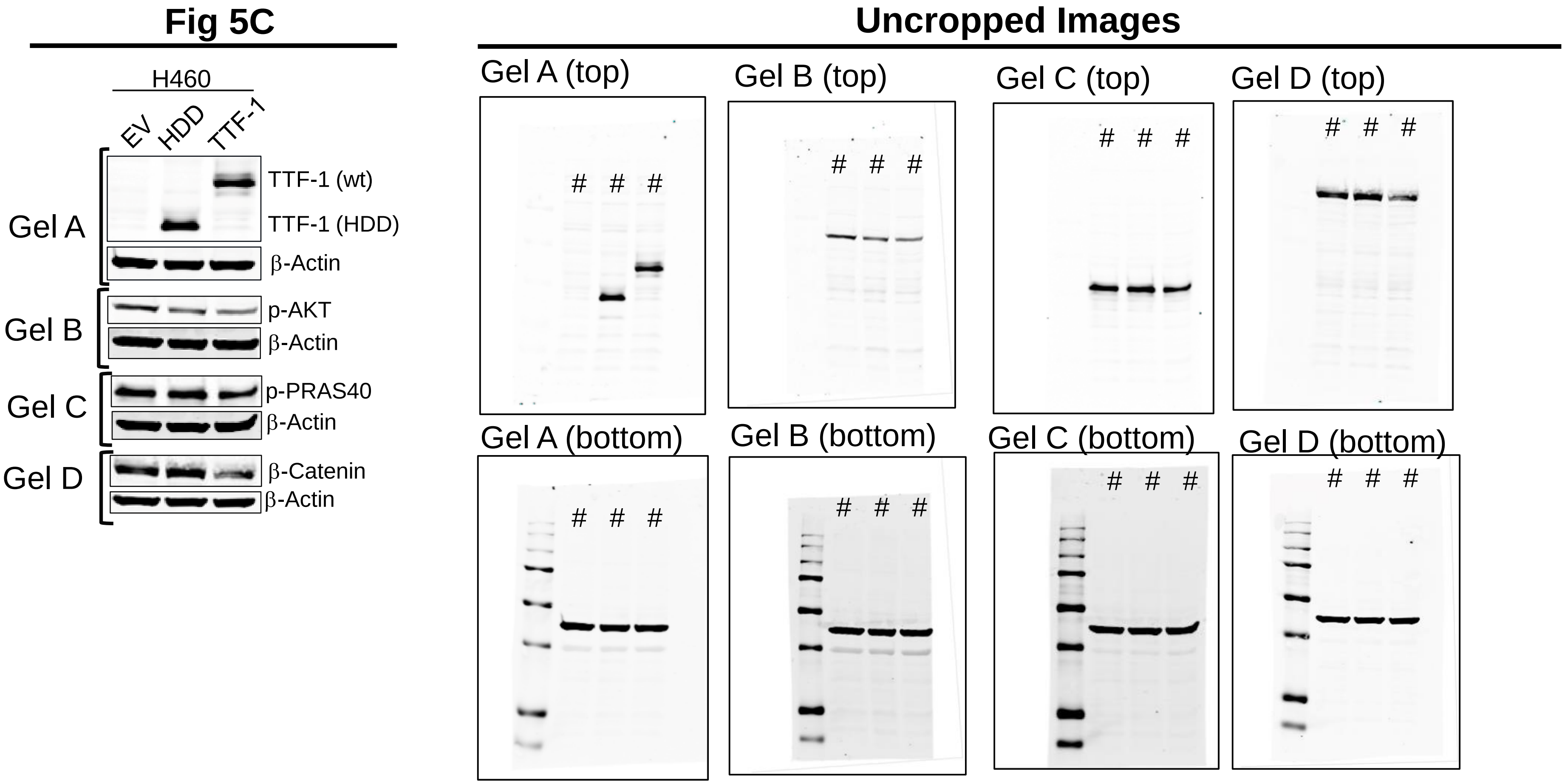

Figure S10. Fig 5C and the original uncropped images. # indicates the lanes containing the cropped bands shown in Fig 5C.

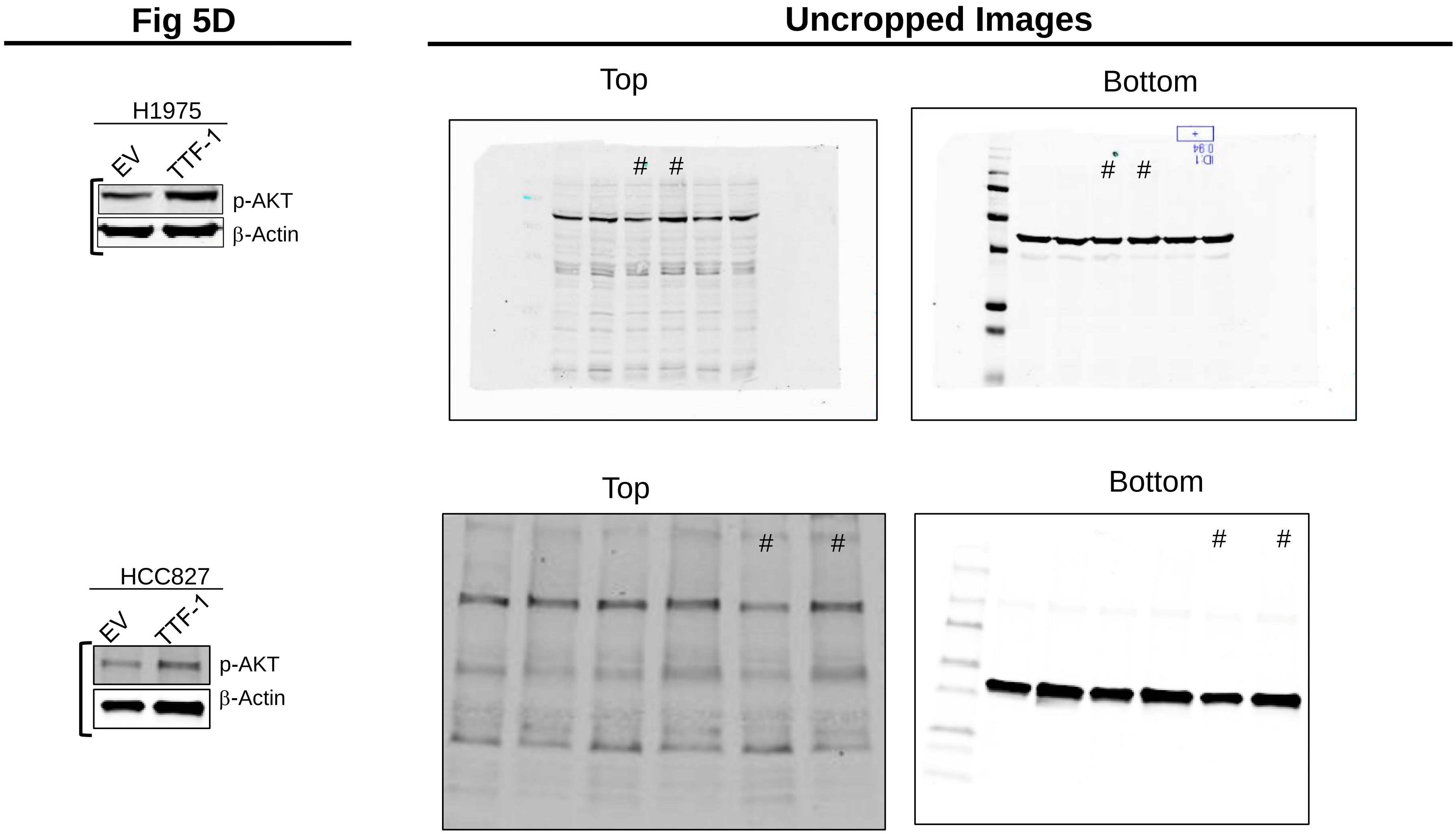

Figure S11. Fig 5D and the original uncropped images. # indicates the lanes containing the cropped bands shown in Fig 5D. The cropped images include one biological replicate whereas the uncropped images include three biological replicates.

Fig 5E

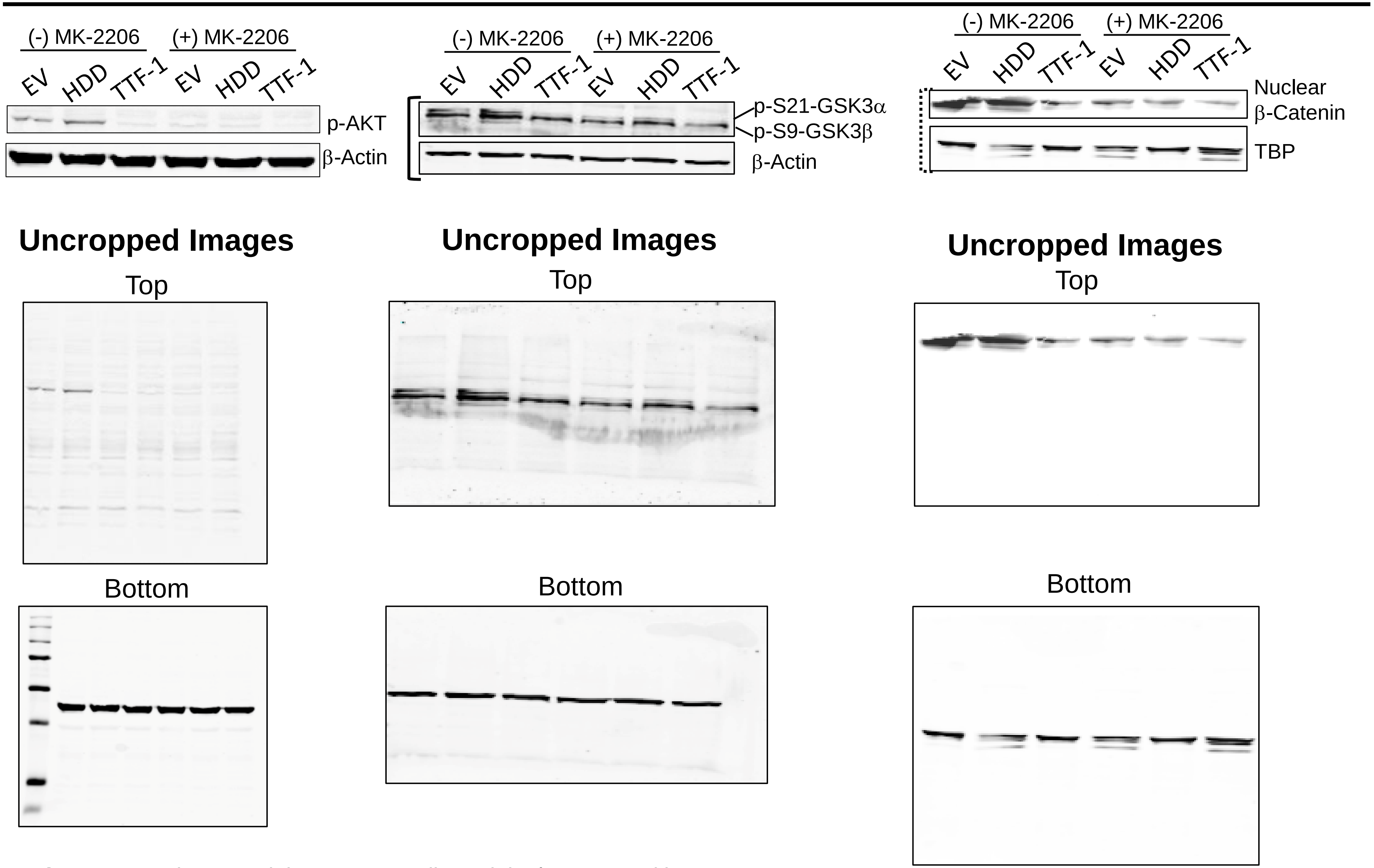

Figure S12. Fig 5E and the corresponding original uncropped images.
